# Utilising the diagnostic criteria of paediatric feeding disorder: Updated findings from a population‐based cohort study

**DOI:** 10.1002/jpn3.70258

**Published:** 2025-11-06

**Authors:** Natalie Raven Morris, Hannah Daw, Lucy Bates, Richard David Horniblow

**Affiliations:** ^1^ The Feeding Trust Birmingham UK; ^2^ Department of Biomedical Sciences, School of Infection, Inflammation, and Immunology, University of Birmingham, Edgbaston College of Medicine and Health Birmingham UK

**Keywords:** clinical predictors, diagnostic framework, feeding difficulties, multidisciplinary care

## Abstract

**Objectives:**

Paediatric feeding disorder (PFD) is highly prevalent but frequently underdiagnosed in the United Kingdom. Existing diagnostic criteria provide a valuable framework for identifying and managing PFD. However, inconsistent use of these criteria results in under‐reporting of prevalence and presentation. This study aims to expand current clinical evidence and evaluate the diagnostic framework to better reflect the real‐world prevalence of PFD in United Kingdom practice. By reviewing and applying these criteria, we aim to enhance diagnostic accuracy, support early identification and improve care implementation.

**Methods:**

This observational cohort study enrolled 51 patients with PFD. Patients were examined against the current PFD diagnostic criteria and characterised according to the four areas of impairment for diagnosis of PFD, identifying the prevalence of medical, nutritional, psychosocial and feeding skill conditions.

**Results:**

The most prominent clinical features in patients with PFD were medical and skill‐based. All PFD patients had conditions associated with skill‐based difficulties; 96% presented with a medical condition, 51% had a psychosocial condition, and 20% had a diagnosed nutritional deficiency. There was a high incidence of multiple, co‐occurring conditions.

**Conclusion:**

This is the first study to report the high prevalence of skill‐based deficits in PFD, along with the type and frequency of clinical features observed in affected patients. Based on these findings, we propose a clarification of the diagnostic criteria, with greater emphasis on medical and skill‐based factors. This study underscores the importance of a comprehensive, multidisciplinary assessment of feeding, incorporating a thorough evaluation of feeding skills and the functional impact of medical conditions on feeding development.

## Abstract (highlights)

**What is Known:**

- Paediatric feeding disorder (PFD) affects an estimated 2.5%–3.5% of children under five, with prevalence reaching up to 80% in those with developmental disabilities.
- Understanding the interplay of presenting conditions in PFD is critical for identifying at‐risk children, enabling early diagnosis and treatment, and mitigating poor growth and related complications.

**What is New:**

- Skill‐based feeding deficits are the most observed features in PFD but remain under‐reported in paediatric populations.
- Neurodevelopmental and gastrointestinal conditions are the most frequent medical comorbidities.
- PFD typically involves multiple coexisting conditions, complicating accurate diagnosis.
- This study offers clinical evidence to inform diagnostic refinement, aiding differentiation between PFD and other feeding disorders and enhancing clinical decision‐making.

## Graphical abstract

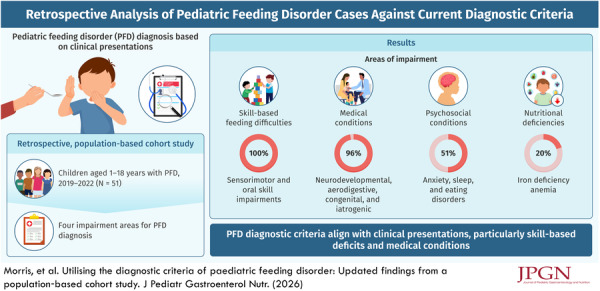

## 1 INTRODUCTION

Feeding difficulties are reported in approximately 25% of typically developing children and up to 80% of those with developmental disabilities.^1^, ^2^, ^3^ However, precise prevalence estimates are hindered by inconsistent definitions and classification of feeding problems.^4^ The aetiology of feeding difficulties is complex and multifactorial, involving disruptions in physiological, neurological, behavioural and environmental mechanisms that underpin feeding behaviour. These disruptions can interfere with the acquisition and development of feeding skills, resulting in impaired oral intake. Affected children frequently present with co‐occurring medical, nutritional and developmental conditions.

In response to the need for a unified diagnostic framework, paediatric feeding disorder (PFD) was formally recognised in the US International Classification of Diseases (ICD) following the publication of a consensus paper in 2019.^5^ This study introduced a standardised definition and conceptual framework for PFD, aimed at improving diagnosis and guiding clinical management. PFD is defined by dysfunction in one or more of four core domains: medical, nutritional, feeding skills and psychosocial.^6^ These domains interact in complex ways and contribute to the persistence and heterogeneity of feeding difficulties in paediatric populations. Despite its inclusion in the US ICD, PFD is not currently recognised in ICD‐11. This lack of formal classification contributes to underdiagnosis in the United Kingdom, where clinicians may instead use broader, nonspecific terms such as ‘feeding difficulties’ or ‘sensory feeding’, leading to variable recognition and delayed intervention.

A deeper understanding of the relationship between PFD and its associated comorbidities is crucial for enhancing early identification and intervention. Mapping these associations may also shed light on the mechanisms that maintain feeding difficulties over time. The present study aims to evaluate the prevalence and types of presenting conditions in children diagnosed with PFD and to explore the frequency and patterns of co‐occurrence of these conditions within a clinical cohort.

## 2 METHODS

### 2.1 Ethics statement

This study was conducted according to the guidelines of the Declaration of Helsinki and approved by the University of Birmingham Science, Technology, Engineering and Mathematics Ethics Committee (ERN_2442 April 2024).

### 2.2 Study setting and data source

This retrospective, population‐based cohort study included 58 children and young people (CYP) aged 1–18 years diagnosed with PFD by the feeding trust (TFT) between 2019 and 2022. TFT is a UK‐based, therapist‐led charity comprising a multidisciplinary team (MDT) of paediatric dietitians, speech and language therapists (SLTs) and occupational therapists (OTs).

Participants were referred by parents, NHS professionals, or social care services due to severe feeding difficulties and travelled from across the United Kingdom to attend TFT clinics. Referrals were triaged based on the severity of feeding difficulties, and all included children met the following referral criteria:

I) Persistent feeding difficulties lasting over 12 months and unresponsive to first‐ and second‐line support from local services.
II) Markedly restricted dietary variety, defined as the acceptance of fewer than 10 different foods.
III) Significant functional impact, including disruption to mealtime participation, nutritional intake or family routines.

PFD was diagnosed by the MDT using the International Statistical Classification of Diseases and Related Health Problems, 10th Revision (ICD‐10) framework.

Clinical data were extracted from TFT electronic health records in 2022 and included age, sex, neurodevelopmental and psychiatric diagnoses, anthropometric data, perinatal and medical history and feeding history. Data extraction was performed by trained reviewers using standardised protocols to minimise misclassification or omission.

### 2.3 Study inclusion criteria

For inclusion in this study, CYP were required to have a multidisciplinary diagnosis of PFD made by TFT at the time of their clinical assessment, using the Goday et al. diagnostic criteria. Eligible participants were aged between 1 and 18 years, from any demographic background, with any comorbidity status (including none, single or multiple comorbidities), and with any anthropometric measurements. Sufficient clinical assessment data across at least three of the four PFD diagnostic domains (medical, nutritional, feeding skill and/or psychosocial) were required for inclusion. This approach ensured a clinically diverse cohort representative of real‐world presentations.

Incomplete data were identified in seven patients, who either failed to attend the assessment or did not meet the diagnostic criteria for PFD, and these cases were excluded from the analysis.

### 2.4 Retrospective application of current diagnostic criteria

Assessment data from the PFD cohort was retrospectively analysed against the current diagnostic criteria.^5^ The four areas of impairment for diagnosis of PFD based on the Diagnostic Criteria Case Report Form^7^ are summarised in Table S1. Utilising these areas for diagnosis ensured consistent criteria were applied across all CYP. Given the retrospective nature of the study and reliance on prior clinical assessments, there is potential for bias due to variability in diagnostic practices and documentation among healthcare professionals.

### 2.5 Assessment of the four PFD domains

Each CYP underwent a multidisciplinary assessment by a team of specialist paediatric clinicians, including OTs, SLTs and paediatric dietitians. Assessments were conducted according to TFT standardised clinical protocol, informed by the Goday et al. diagnostic framework. While no single validated tool exists that covers all four PFD domains comprehensively, a combination of clinical assessments and structured observations was used:

i) *Medical domain:* Based on documented medical history, diagnoses from referring health professionals (e.g., paediatricians, gastroenterologists), and medical records reviewed by clinicians.
ii) *Nutritional domain:* Evaluated by paediatric dietitians through dietary recall, growth charts and review of clinical nutrition history.
iii) *Feeding skill domain:* Assessed by speech and language and OTs: Review of developmental history; clinical observations of feeding, functional skills and development;
iv) *Psychosocial domain:* Informed by structured interviews with parents/carers, review of referral documentation and behavioural observations during mealtimes. Diagnosed mental health conditions were recorded where formally identified by referring professionals.

### 2.6 Condition frequency in PFD patients

A comorbid condition was defined as any diagnosis made at any age by a relevant healthcare professional. Individual diagnoses were grouped into broader categories of impairment based on the *Diagnostic Criteria Case Report Form*.^7^ These categories are summarised in Table 1. Each patient was counted once per category, regardless of the number of diagnoses within that group (e.g., a child with both anxiety and depression was counted once under mental health conditions).

**Table 1:** Condition categories in PFD.

**Medical**	
Aerodigestive: gastrointestinal/airway/pulmonary	
	Constipation
	IBS
	NEC
	Reflux and/or vomiting
	Chronic lung disease
Oral/nasal/pharyngeal disorders	
	Craniofacial condition
Congenital and other heart disease	
	Heart condition
	Congenital/chromosomal
Neurodevelopmental condition	
	ASC
	ADHD
	Cerebral palsy
	Down syndrome
	Learning difficulties/developmental delay
	Speech/language/communication delay
Iatrogenic	
	Prematurity
Other medical (unclassified—not included in Diagnostic Criteria Case Report Form)	
	Coeliac disease
	CMPA
	Egg/peanut/soya allergy
	Hay fever
	Eczema
	Seizures
**Nutritional**	
Iron deficiency anaemia	
Vitamin D deficiency	
**Feeding skill**	
Under/over responsive sensory processing (sensory modulation)	
Impairments in motor functioning (sensory based motor difficulties)	
Oral skill difficulties (inefficient/delayed oral feeding)	
**Psychosocial**	
Anxiety	
Depression	
Eating disorder	
Sleep disorders	

Abbreviations: ADHD, attention deficit hyperactivity disorder; ASC, autism spectrum condition; CMPA, cow's milk protein allergy; IBS, irritable bowel syndrome; NEC, necrotising entercolitis; PFD, paediatric feeding disorder.

### 2.7 Body mass index (BMI) analysis and categorisation

Analysis of BMI was based on guidance from the Royal College of Paediatrics and Child Health (RCPCH). Overweight was classed as BMI >91st centile, ‘healthy’ BMI between the 2nd and the 91st centile and underweight BMI <2nd centile.

### 2.8 Data analysis and statistical methods

Relative frequencies were calculated for each diagnostic category, for individual comorbid conditions (whether occurring alone or in combination), and for the total number of comorbidities per patient. The number of comorbid conditions was treated as a continuous variable in all analyses. Data were analysed using Microsoft Excel with graphs created using GraphPad Prism. All subjects were anonymised throughout the analyses. Where possible, independent verification of extracted data was conducted by a second reviewer.

Since this study is descriptive and presents raw counts of comorbid conditions in patients with PFD. No statistical analyses were conducted, and no adjustments were made for confounding variables. No subgroup or interaction analyses were performed.

## 3 RESULTS

### 3.1 Patient population

The study included 51 CYP: 30 male and 21 female. The mean age was 6 years (SD = 3.6), ranging from 1 to 16 years, with the majority under 9 years of age (Figure S1).

### 3.2 Patient BMI

The majority of CYP (84%) had a BMI within the recommended reference range, compared to 89% in the general paediatric population based on RCPCH centile charts (Table S2).

A small proportion had a BMI below the 2nd centile (6%) or above the 91st centile (6%), while BMI could not be assessed in 4% of CYP due to age. Notably, all CYP with a BMI outside the healthy centile range were under 7 years of age (Figure S2).

### 3.3 Condition prevalence

**Figure 1:**
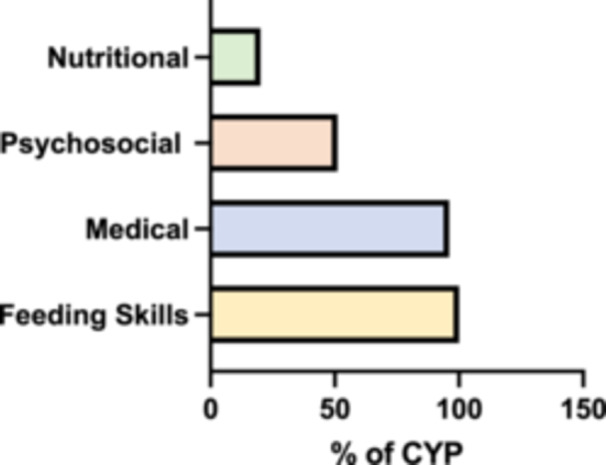
Prevalence of impairment across the four PFD domains. The proportion (%) of CYP presenting with difficulties in each of the four domains: skill‐based, medical, psychosocial and nutritional. CYP, children and young people; PFD, paediatric feeding disorder.

**Figure 2:**
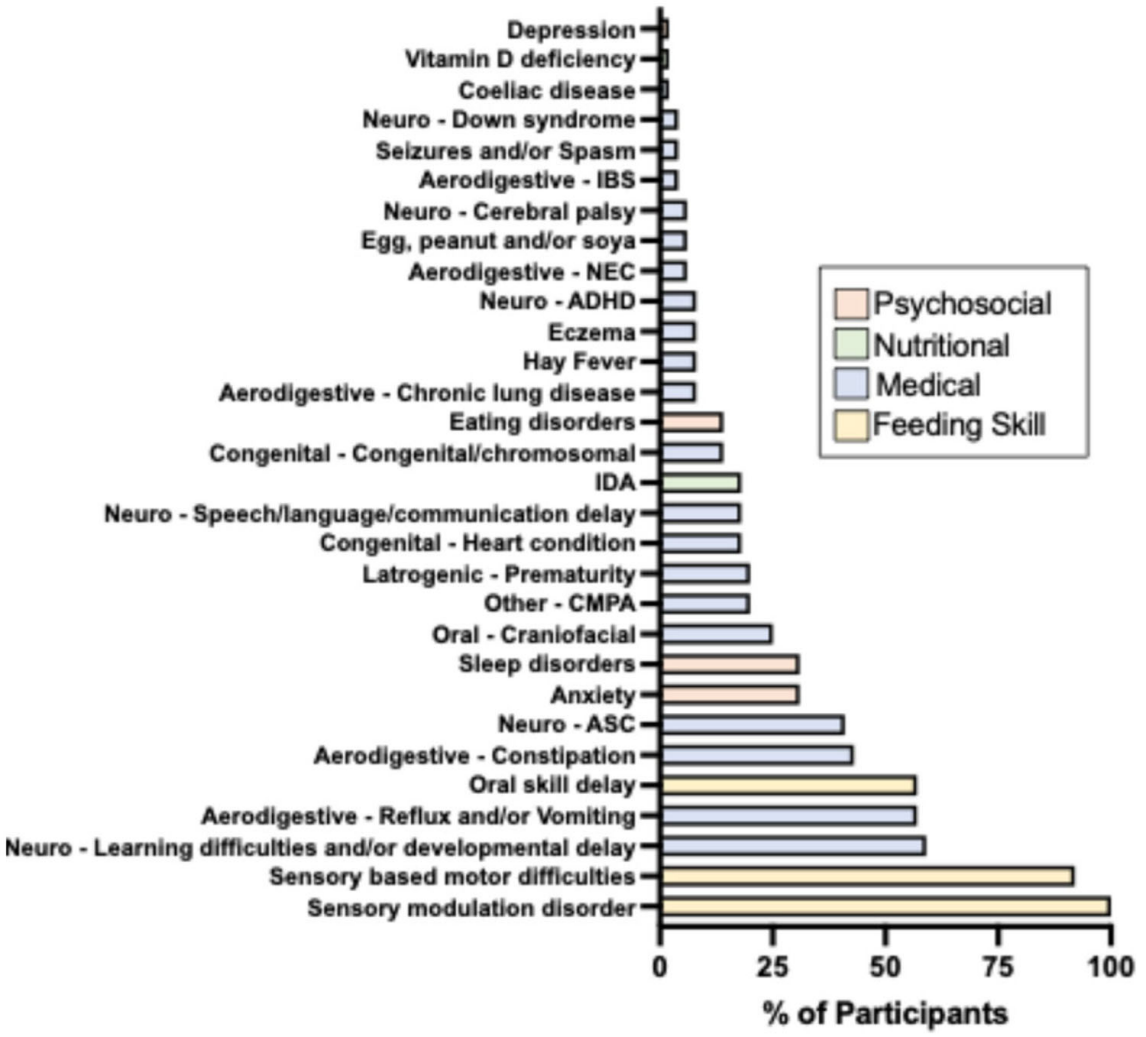
Frequency of individual comorbid conditions within each impairment domain. Frequency (% of total cohort) of the number of CYP diagnosed with specific conditions grouped by domain (e.g., neurodevelopmental, gastrointestinal, mental health), highlighting the overlap and distribution of comorbidities. ADHD, attention deficit hyperactivity disorder; ASC, autism spectrum condition; CMPA, cow's milk protein allergy; CYP, children and young people; IDA, iron deficiency anaemia; IBS, irritable bowel syndrome; NEC, necrotising entercolitis.

Across the four domains of impairment, all CYP (100%) exhibited skill‐based feeding difficulties. Medical conditions were present in 96% of the cohort, while 51% had a psychosocial diagnosis, and 20% had a documented nutritional deficiency (Figure 1). A detailed breakdown of the frequency of individual conditions within each domain is provided in Figure 2.

#### 3.3.1 Medical conditions

Neurodevelopmental disorders were the most prevalent comorbidities in the cohort. Over half of the CYP had documented learning difficulties and/or developmental delay, and 41% had a diagnosis of autism spectrum condition (ASC).

Aerodigestive conditions were also common, particularly gastrointestinal issues. Reflux and/or vomiting affected 57% of CYP, while 43% experienced constipation. Allergies were included within this category, with cow′s milk protein allergy (CMPA) being the most frequently reported.

Other medical conditions included craniofacial abnormalities (25%), congenital or chromosomal disorders (14%) and cardiac abnormalities (18%). In addition, nearly one‐quarter of the cohort had experienced complications resulting from medical interventions, such as prematurity, categorised here as ‘iatrogenic’ factors.

#### 3.3.2 Nutritional conditions

As a criterion for study inclusion, all CYP had inadequate dietary diversity (fewer than 10 accepted foods). Additionally, 20% of the cohort had a diagnosed nutritional deficiency, predominantly iron deficiency anaemia (IDA).

#### 3.3.3 Feeding skill conditions

All CYP exhibited sensory over‐ or under‐responsivity (sensory modulation difficulties). Sensory‐based motor difficulties were highly prevalent, affecting 92% of participants, while oral motor skill impairments were observed in just over half of the cohort.

#### 3.3.4 Psychosocial conditions

Among prediagnosed psychosocial conditions, anxiety and sleep disorders were the most common (both 31%), followed by eating disorders, specifically avoidant/restrictive food intake disorder (ARFID), at 14%.

#### 3.3.5 Comorbid conditions

There was substantial overlap in comorbidities, with 65% of CYP having six or more co‐occurring conditions, while fewer than 15% had fewer than five (Figure S3).

## 4 DISCUSSION

A substantial proportion (78%) of the cohort had a neurodevelopmental condition, markedly higher than the estimated 6% in the general paediatric population.^8^ Learning difficulties and developmental delay were the most frequently reported, consistent with evidence that children with feeding difficulties are up to four times more likely to have developmental delays.^9^ Additionally, 41% were diagnosed with ASC, aligning with prevalence rates reported in comparable cohorts.^10^ Given the strong association between PFD and neurodevelopmental disorders, routine screening for feeding difficulties in these populations may facilitate earlier diagnosis and intervention.

Gastrointestinal dysfunction was also highly prevalent, with 57% of CYP experiencing reflux or vomiting, and 43% presenting with constipation. These conditions are known to disrupt feeding behaviours and limit dietary intake^11^ and were notably more common in this cohort than in the general paediatric population.^12^, ^13^ Food allergies affected 26% of participants, predominantly CMPA, which is associated with aversive feeding experiences and may contribute to the development or maintenance of PFD.^14^

Cardiac and craniofacial anomalies were present in 40% of the cohort, consistent with previous findings.^15^, ^16^ Additionally, 20% of CYP were born prematurely, a well‐established risk factor for persistent feeding difficulties.^17^ These findings highlight the importance of early, multidisciplinary feeding support for high‐risk medical populations.

Consistent with findings by Junqueira et al., 84% of the cohort had a BMI within the reference range, highlighting the limitation of BMI as a standalone marker of nutritional adequacy in PFD.^18^ All participants demonstrated reduced dietary diversity, frequently omitting entire food groups—an issue that can compromise micronutrient intake even in the absence of underweight status.

Despite these restrictive patterns, only 20% had a diagnosed nutritional deficiency, with iron deficiency being the most common. This is particularly relevant, as iron deficiency is known to impair appetite and exacerbate feeding difficulties.^19^ Given the critical role of micronutrients in growth, cognitive function and overall development,^20^ routine nutritional assessment (including dietary diversity and micronutrient status) should be a key component of PFD management.

Feeding skill development is closely linked to sensory processing, which governs how the central nervous system perceives, integrates and responds to sensory input. Disruptions in this system can profoundly affect feeding behaviours, and in our cohort, all children exhibited sensory processing challenges.

All CYP (100%) had sensory modulation differences, including over‐responsiveness (e.g., sensory avoidance, hypersensitivity) and under‐responsiveness (e.g., sensory seeking, poor registration). Additionally, 92% presented with sensory‐motor difficulties, particularly in bilateral integration and somatodyspraxia, affecting postural control and coordination. These issues made maintaining an upright posture during meals difficult, often leading to fatigue and making feeding physically demanding.

Oral motor difficulties were identified in 57% of the cohort. Parents commonly reported issues such as delayed texture progression, chewing difficulties, food pocketing, gagging and spitting. Notably, none had been previously diagnosed, supporting reports that sensory‐motor challenges in feeding often go undetected.^21^

Typical oral sensory‐motor development requires coordinated input from taste, tactile and proprioceptive systems. However, early medical experiences such as nasogastric feeding, reflux or food allergies can disrupt the usual exposure–experience–skill development trajectory during critical sensorimotor and neophobic stages.

Our findings reinforce that feeding skill acquisition is not limited to early childhood. While previous research has focused primarily on children under 9,^2^, ^6^, ^22^ difficulties in our cohort persisted into adolescence (up to age 16). Emerging evidence suggests that children with complex medical and developmental needs may continue to refine feeding skills into preadolescence (11–12 years).^23^ These findings underscore the importance of sustained feeding interventions beyond the early years.

Nearly one‐third of our cohort had sleep disorders and anxiety. Poor sleep has been linked to unhealthy eating habits and appetite dysregulation,^24^ making sleep assessment a critical part of PFD management.

Mental health conditions were diagnosed in 37% of the CYP, twice the prevalence seen in the general UK paediatric population.^11^ Anxiety was most common, though its relationship with PFD is complex, it may be a contributing factor, a consequence or both. Early recognition is essential, as fear of choking, vomiting or food textures may be core components of feeding difficulties.^5^

Eating disorders were found in 14% of the CYP, specifically ARFID. Although PFD and ARFID share overlapping features, recent research suggests that the two conditions can co‐exist rather than being mutually exclusive.^25^ ARFID is a psychiatric condition involving persistent food restriction with nutritional or psychosocial consequences whereas PFD (as defined by Goday et al.), involves impaired oral intake due to dysfunction in medical, nutritional, feeding skill, or psychosocial domains. The two can co‐occur, and CYP often present with early feeding skill delays and later develop avoidant behaviours and anxiety around food, meeting criteria for both PFD and ARFID. Recognising this distinction is important for ensuring appropriate, needs‐based, individualised interventions.

By definition, PFD involves impairments across one or more functional domains.^5^ This complexity was evident in our cohort, with 65% of children presenting with six or more comorbid conditions. Such a high level of multimorbidity presents significant clinical challenges, particularly within healthcare systems typically organised around single‐diagnosis care pathways. This structural limitation can lead to fragmented services and suboptimal outcomes.^26^

Our findings underscore the critical importance of MDT involvement in the management of PFD. Coordinated input from paediatricians, SLTs, dietitians, OTs and mental health professionals is essential to meet the multifaceted needs of this population. This is particularly important in complex cases with multiple co‐occurring conditions, where care must be tailored to address the specific combination of medical, nutritional, skill‐based and psychosocial challenges to optimise outcomes.

In addition to the clinical demands, multimorbidity also places a considerable burden on caregivers. Parents of children with feeding difficulties report higher stress levels than those caring for children without such challenges.^2^ Emotional strain, time constraints and the complexity of navigating care can be overwhelming. Yet caregivers are pivotal to the success of feeding therapy. Supporting parents through training, counselling and respite care may enhance treatment adherence and outcomes by empowering them to implement therapeutic strategies effectively at home.

This study has several limitations, including its small sample size, retrospective observational design, and the absence of a control group. While we successfully documented the prevalence of comorbidities in children with PFD, the lack of a comparison group without PFD limits our ability to assess the relative significance of these findings. As a retrospective observational study, we could not establish causality, particularly regarding the association between sensory processing difficulties and PFD. Our findings suggest a strong relationship, but the direction and nature of this link remain uncertain.

Diagnostic consistency presents another limitation. Comorbid health conditions were identified based on prior assessments conducted by various external health professionals, which introduces variability in diagnostic criteria and reporting. This introduces the potential for diagnostic bias, as the criteria and rigour applied by different professionals may vary, influencing the consistency and accuracy of recorded comorbidities. Furthermore, as the data were drawn from a clinical cohort, our findings may not reflect the full spectrum of children with PFD, particularly those who remain undiagnosed or have not accessed specialist services. The exclusion of seven patients with incomplete data may have introduced a selection bias, as these cases may have represented individuals with more complex or atypical presentations not captured in the final analysis.

Future research should address these gaps through prospective, multicentre studies with larger and more diverse samples, including well‐matched control groups. Longitudinal designs could also help clarify causal relationships between sensory, medical, and skill‐based impairments and the onset or persistence of PFD. To strengthen diagnostic consistency in the field, there is a pressing need for PFD to be included within the ICD‐11 and a professional consensus on standardised assessment tools that encompass all four functional domains of PFD. At present, no single instrument provides comprehensive coverage across these domains. In the absence of such a tool, we utilised The Feeding Trust′s structured multidisciplinary protocol, which operationalises the Goday framework within clinical practice. Future work should aim to develop and validate such tools.

Our findings strongly support the need for targeted interventions that address sensory and motor deficits in children with PFD. Given the high prevalence of sensory modulation and sensory‐based motor difficulties in this cohort, feeding interventions should include individualised sensory assessments to guide therapy. Interventions might focus on improving postural control, oral‐motor coordination and sensory regulation to support skill acquisition and functional mealtime participation. Future studies should evaluate the effectiveness of these approaches through longitudinal, mixed‐methods designs using validated outcome measures (such as feeding competence, nutritional intake and caregiver‐reported quality of life).

We utilised the Goday et al. PFD diagnostic case report form to structure our analysis. However, we found its interpretation of sensory processing to be narrow, focusing primarily on oral‐pharyngeal responsiveness and motor function. Our findings highlight a broader impact of sensory differences on feeding development, including mealtime alertness, posture, coordination and self‐feeding abilities. In light of this, we recommend that sensory processing differences be formally recognised within the skill domain of the PFD diagnostic framework.

## 5 CONCLUSIONS

This study provides clinical evidence linking PFD to sensory processing difficulties, neurodevelopmental conditions, gastrointestinal disorders and oral‐motor skill delays. These factors may serve as key predictors and highlight the need for multidisciplinary assessment.

Our use of the Goday framework identified the *medical* and *skill‐based* domains as the most significant contributors to PFD. Notably, undiagnosed oral‐motor skill deficits were highly prevalent. We propose that differences across all eight sensory systems be formally integrated into the skill domain of the PFD diagnostic framework.

Despite normal BMI in most children, restricted dietary diversity was common, indicating a risk of micronutrient deficiency and reinforcing that BMI alone is not a reliable nutritional marker in PFD. These findings support the complex, multidomain nature of PFD and the necessity for coordinated and multidisciplinary care. Further research should focus on refining the diagnostic criteria, standardising assessment tools and enabling earlier identification and intervention.

## CONFLICT OF INTEREST STATEMENT

The Feeding Trust is the UK Charity for Paediatric Feeding Disorders (PFD). Registered charity number: 1205834. Natalie Raven Morris and Lucy Bates are Charity directors, and Hannah Daw and Richard David Horniblow are Charity Trustees.

## Supporting information

a wide frequency distribution across the ages of cyps in the study. age distribution of cyp in the study. histogram bars indicate the number of participants at each age (in years).

bmi distribution among the patient cohort. bars show the number of cyp with bmi centiles classified as within the healthy reference range, underweight ( < 2nd centile), or overweight ( > 91st centile).

histogram bars represent the number of cyp and their total number of conditions.

impairment areas for diagnosis of pfd according to the diagnostic criteria case report.

bmi ranges of the pfd patient cohort; values given as relative frequency (percentage).
