# Supplementary figures and images for: Utilising the diagnostic criteria of paediatric feeding disorder: Updated findings from a population‐based cohort study
Source: J Pediatr Gastroenterol Nutr. 2025 Nov 6;82(1):33–41. doi: 10.1002/jpn3.70258 (PMC12780484; doi:10.1002/jpn3.70258)

## Slide 1
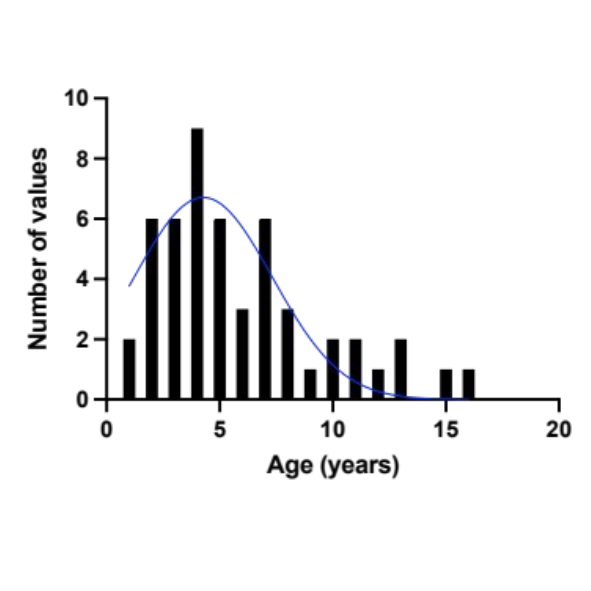

Supplement: Supplementary file 1 — a wide frequency distribution across the ages of cyps in the study. age distribution of cyp in the study. histogram bars indicate the number of participants at each age (in years). [file JPN3-82-33-s003.pptx]

## Slide 1
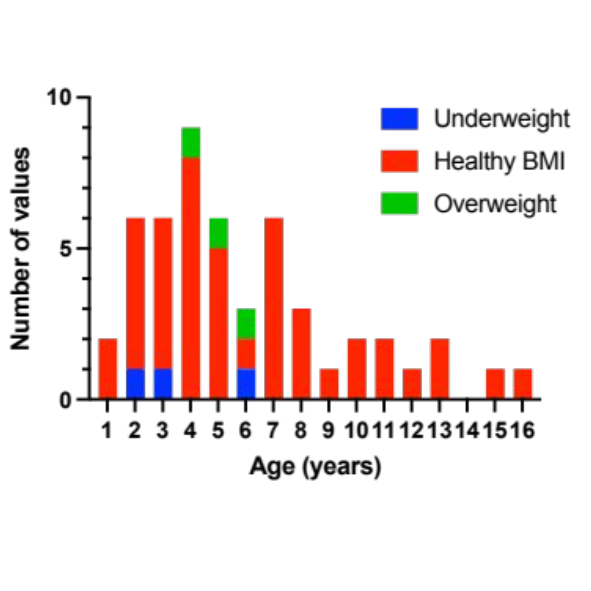

Supplement: Supplementary file 2 — bmi distribution among the patient cohort. bars show the number of cyp with bmi centiles classified as within the healthy reference range, underweight ( < 2nd centile), or overweight ( > 91st centile). [file JPN3-82-33-s002.pptx]

## Slide 1
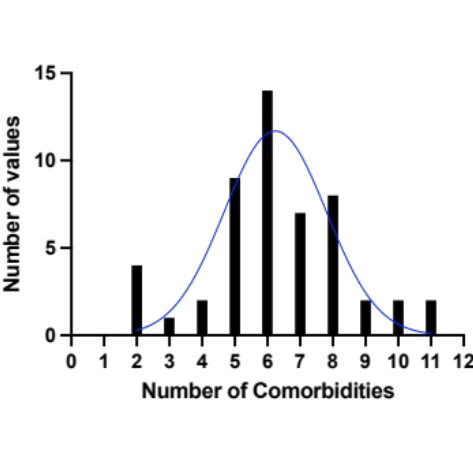

Supplement: Supplementary file 3 — histogram bars represent the number of cyp and their total number of conditions. [file JPN3-82-33-s005.pptx]
