# Supplementary material for: Utilising the diagnostic criteria of paediatric feeding disorder: Updated findings from a population‐based cohort study
Source: J Pediatr Gastroenterol Nutr. 2025 Nov 6;82(1):33–41. doi: 10.1002/jpn3.70258 (PMC12780484; doi:10.1002/jpn3.70258)
Supplement: Supplementary file 4 — impairment areas for diagnosis of pfd according to the diagnostic criteria case report. [file JPN3-82-33-s004.docx]

| **Medical** | **Nutritional** | **Feeding Skill** | **Psychosocial** |
| --- | --- | --- | --- |
| Aerodigestive (including airway and gastrointestinal)  Oral/nasal/pharyngeal disorders | Micro-nutrient | Under/over responsive sensory processing | Mental health diagnosis (anxiety, depression, eating disorder) |
| Congenital and other heart disease | Macro-Nutrient | Impairments in motor functioning | Social (caregiver-child dyad) |
| Neurodevelopmental eg ASC, CP, ADHD | Inadequate dietary diversity | Unsafe / inefficient oral feeding | Environmental (feeding environment, schedules and behaviour) |
| Iatrogenic (hospitalisation, including prematurity) |  | Delayed feeding skills, including self-feeding |  |
