# Supplementary material for: Utilising the diagnostic criteria of paediatric feeding disorder: Updated findings from a population‐based cohort study
Source: J Pediatr Gastroenterol Nutr. 2025 Nov 6;82(1):33–41. doi: 10.1002/jpn3.70258 (PMC12780484; doi:10.1002/jpn3.70258)
Supplement: Supplementary file 5 — bmi ranges of the pfd patient cohort; values given as relative frequency (percentage). [file JPN3-82-33-s001.docx]

|  | **Total sample**  **n (%)** | **Male**  **n (%)** | **Female**  **n (%)** |
| --- | --- | --- | --- |
| Heathy BMI  (2^nd^-91^st^ centile) | 43 (84) | 25 (49) | 18 (35) |
| Low BMI  (<2^nd^ centile) | 3 (6) | 1 (2) | 2 (4) |
| High BMI  (>91^st^ centile) | 3 (6) | 2 (4) | 1 (2) |
